# HATMSC Secreted Factors in the Hydrogel as a Potential Treatment for Chronic Wounds—In Vitro Study

**DOI:** 10.3390/ijms222212241

**Published:** 2021-11-12

**Authors:** Honorata Kraskiewicz, Piotr Hinc, Agnieszka Krawczenko, Aleksandra Bielawska-Pohl, Maria Paprocka, Danuta Witkowska, Isma Liza Mohd Isa, Abhay Pandit, Aleksandra Klimczak

**Affiliations:** 1 Laboratory of Biology of Stem and Neoplastic Cells, Hirszfeld Institute of Immunology and Experimental Therapy, Polish Academy of Sciences, R. Weigla 12, 53–114 Wroclaw, Poland; hincpiotr2@gmail.com (P.H.); agnieszka.krawczenko@hirszfeld.pl (A.K.); aleksandra.bielawska-pohl@hirszfeld.pl (A.B.-P.); maria.paprocka@hirszfeld.pl (M.P.); 2 Laboratory of Medical Microbiology, Hirszfeld Institute of Immunology and Experimental Therapy, Polish Academy of Sciences, 53-114 Wroclaw, Poland; danuta.witkowska@hirszfeld.pl; 3 CÚRAM, SFI Research Centre for Medical Devices, National University of Ireland Galway, H91 W2TY Galway, Ireland; ismaliza84@gmail.com (I.L.M.I.); abhay.pandit@nuigalway.ie (A.P.)

**Keywords:** adipose tissue-derived mesenchymal stem cells, mesenchymal stem cell secretome, MSC secretome, chronic wound, collagen hydrogel

## Abstract

Mesenchymal stem cells (MSCs) can improve chronic wound healing; however, recent studies suggest that the therapeutic effect of MSCs is mediated mainly through the growth factors and cytokines secreted by these cells, referred to as the MSC secretome. To overcome difficulties related to the translation of cell therapy into clinical use such as efficacy, safety and cost, we propose a hydrogel loaded with a secretome from the recently established human adipose tissue mesenchymal stem cell line (HATMSC2) as a potential treatment for chronic wounds. Biocompatibility and biological activity of hydrogel-released HATMSC2 supernatant were investigated in vitro by assessing the proliferation and metabolic activity of human fibroblast, endothelial cells and keratinocytes. Hydrogel degradation was measured using hydroxyproline assay while protein released from the hydrogel was assessed by interleukin-8 (IL-8) and macrophage chemoattractant protein-1 (MCP-1) ELISAs. Pro-angiogenic activity of the developed treatment was assessed by tube formation assay while the presence of pro-angiogenic miRNAs in the HATMSC2 supernatant was investigated using real-time RT-PCR. The results demonstrated that the therapeutic effect of the HATMSC2-produced factors is maintained following incorporation into collagen hydrogel as confirmed by increased proliferation of skin-origin cells and improved angiogenic properties of endothelial cells. In addition, HATMSC2 supernatant revealed antimicrobial activity, and which therefore, in combination with the hydrogel has a potential to be used as advanced wound-healing dressing.

## 1. Introduction

Non-healing diabetic wounds very often lead to leg amputation because at present no satisfactory treatment exists. One of the most promising strategies of the experimental trials in this field is mesenchymal stem cells (MSCs)-based therapy [1]. It is attractive due to the differentiation potential of MSCs, their immunomodulatory properties, and paracrine effects [2]. However, more recently it has been suggested that the use of MSC secretome may be an approach that is better for the treatment of chronic wounds than cell transplantation [3,4,5]. Proteomic analysis of MSC conditioned media (MSC-CM) revealed the presence of several hundred proteins involved in angiogenesis, regulation of apoptosis and immune response [6,7,8,9]. Therefore, the MSC secretome is the most important factor responsible for the healing effect of these cells. Moreover, the application of MSC conditioned medium containing bioactive factors, instead of whole cells, can overcome a number of limitations such as poor survival of transplanted cells, invasiveness, and costs of therapy.

Recently, we have established several highly proliferative, non-tumorigenic human adipose tissue-derived mesenchymal stem cell lines (HATMSC). These include HATMSC1 cell line, where immortalized mesenchymal stem cells of adipose tissue origin (atMSCs) were derived from the venous stasis ulcer patient, HATMSC2 cell line where atMSC were derived from a healthy donor, and two clonal populations named HATMSC2D10 and HATMSC2F10 [10]. All cell lines are capable of secreting a potent angiogenic cocktail that promotes human skin-origin cell proliferation in an in vitro chronic wound model [10].

**Figure 1:**
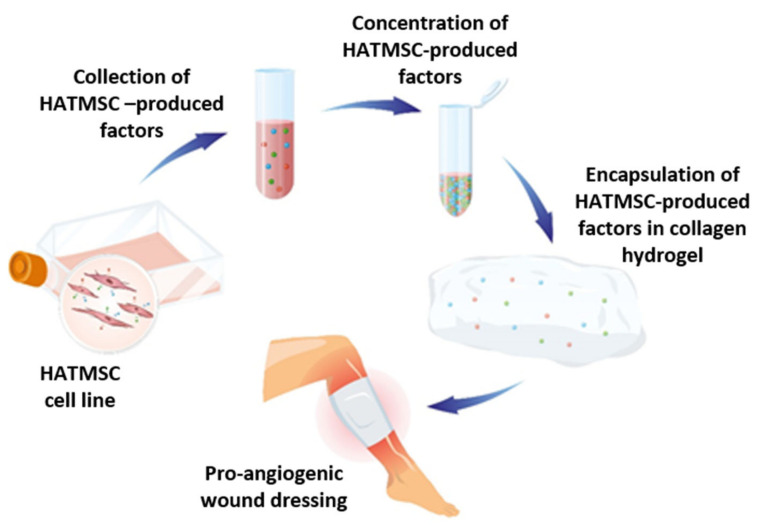
Graphical representation of the research concept. HATMSCs cell line while cultured in vitro secretes a cocktail of therapeutic factors. These factors are collected from the conditioned medium, condensed and incorporated into biomaterial wound dressing which can promote tissue regeneration.

To go further, in this study we evaluate the biological activity of HATMSCs-produced factors following incorporation into collagen hydrogel (Figure 1). For this purpose, we have selected the HATMSC2 cell line, where immortalized atMSC were derived from a healthy donor. Hydrogel-based dressings are increasingly being used in the treatment of chronic wounds because they provide considerable hydration to the wound and reduce the risk of infection. Moreover, hydrogel may also act as a reservoir of a therapeutic factor which is being released in a sustained manner.

To address all the difficulties mentioned that are associated with the use of the whole atMSC, we propose an innovative treatment option for chronic wounds that combines the advantages of cell therapy and hydrogel dressing. This study assesses the hypothesis that the therapeutic effect of the HATMSC2-produced biofactors can be maintained following incorporation into a biomaterial hydrogel, as confirmed in our previous studies on HATMSC2 conditioned medium alone [10]. Chronic wounds are very often accompanied by hypoxia, therefore, to mimic the proper microenvironment of the chronic wound, experiments on the influence of bioactive factors released from hydrogel on the wound healing process, especially on cell proliferation, were carried out under conditions of reduced oxygen concentration.

It has also been shown that in the MSC secretome, apart from regenerative factors, a variety of antimicrobial peptides can be found [11,12]. Having in mind that chronic wounds are often accompanied by bacterial infections [13] which increase the risk for amputation [14], an additional pilot study demonstrating antibacterial activity of the HATMSCs supernatants has been carried out. Our results have shown that the developed hydrogel containing HATMSC2 supernatant characterizes a high multi-approach pro-regenerative potential that can be used for wound dressing preparation.

## 2. Results

### 2.1. Hydrogels Are Not Toxic to Human Skin Origin Cells

**Figure 2:**
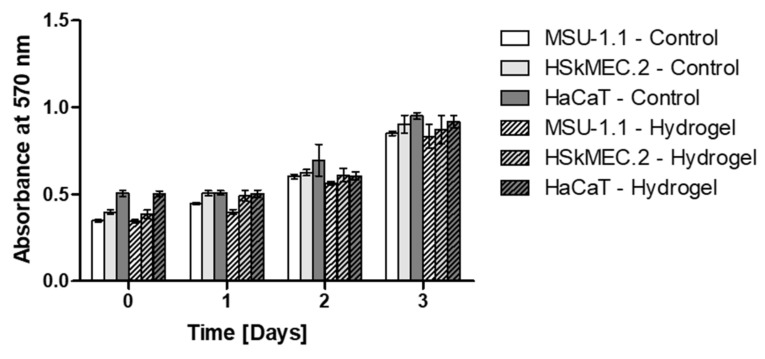
In vitro assessment on biocompatibility of the hydrogel measured by MTT assay. Metabolic activity of MSU-1.1, HSkMEC.2 and HaCaT cells cultured in the presence of hydrogel was measured on day 0, 1, 2 and 3. Untreated cells were used as controls. Data represent mean ± SEM, *n* = 3; no significant differences were observed between hydrogel-treated cells and untreated controls.

The potential cytotoxicity of the designed hydrogel dressing to human fibroblasts (MSU-1.1), endothelial cells (HSkMEC.2) and keratinocytes (HaCaT) was studied by examination of cell metabolic activity cultured in the presence or absence (controls) of hydrogel spheres. For all three tested cell lines, a gradual increase in metabolic activity can be observed, reaching the maximum on the last, third day of the experiment (Figure 2). There were no statistically significant differences between the cells cultured in the presence of the hydrogels and the control groups at any time point. This indicates that the designed hydrogel did not affect the viability of tested cell lines cultured in the standard conditions for up to three days.

### 2.2. Degradation of Hydrogels

**Figure 3:**
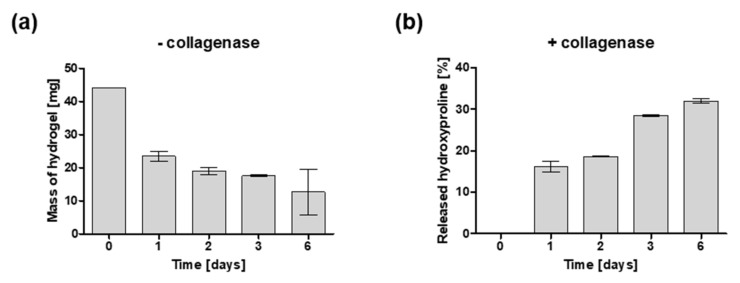
Degradation profile of collagen hydrogels. (**a**) Hydrolytic degradation measured by mass of hydrogel samples incubated in PBS without collagenase enzyme at 37 °C. (**b**) Enzymatic degradation measured by hydroxyproline containing peptides found in the degradation buffer where samples were incubated at 37 °C in the presence of collagenase (25 CDU). Data represent mean ± SEM, *n* = 3.

The prepared collagen hydrogels were exposed to the solution of collagenase in PBS buffer (enzyme-mediated degradation) and PBS buffer alone (hydrolytic degradation) at 37 °C to study the degradation over time for six days. When gels were incubated in PBS buffer without collagenase, a rapid decrease in the mass of hydrogel was observed between day 0 and day 1 (44.14 mg vs. 23.5 mg, respectively). Then, in the following four days, a gradual decrease in hydrogel mass was seen reaching approximately one third of the initial mass on day 6 (12.65 mg) (Figure 3a). In the presence of collagenase, mass degradation could not be carried out within the desired time frame because the spheres were not visible following one day of enzymatic degradation which made weighing impossible. For this reason, the degradation profile in the presence of collagenase was evaluated by the measurement of hydroxyproline content in the degradation buffer (Figure 3b). On day 0, no hydroxyproline was detected in the degradation buffer. However, following one day of study, the hydroxyproline content in the degradation buffer was 16 percent of the initial concentration. In the following days, there was a moderate increase in hydroxyproline concentration reaching 32 percent on day 6. No hydroxyproline was detected in the release buffer without collagenase at any time point.

### 2.3. Bioactivity of Hydrogel-Released HATMSC2-Originated Trophic Factors

**Figure 4:**
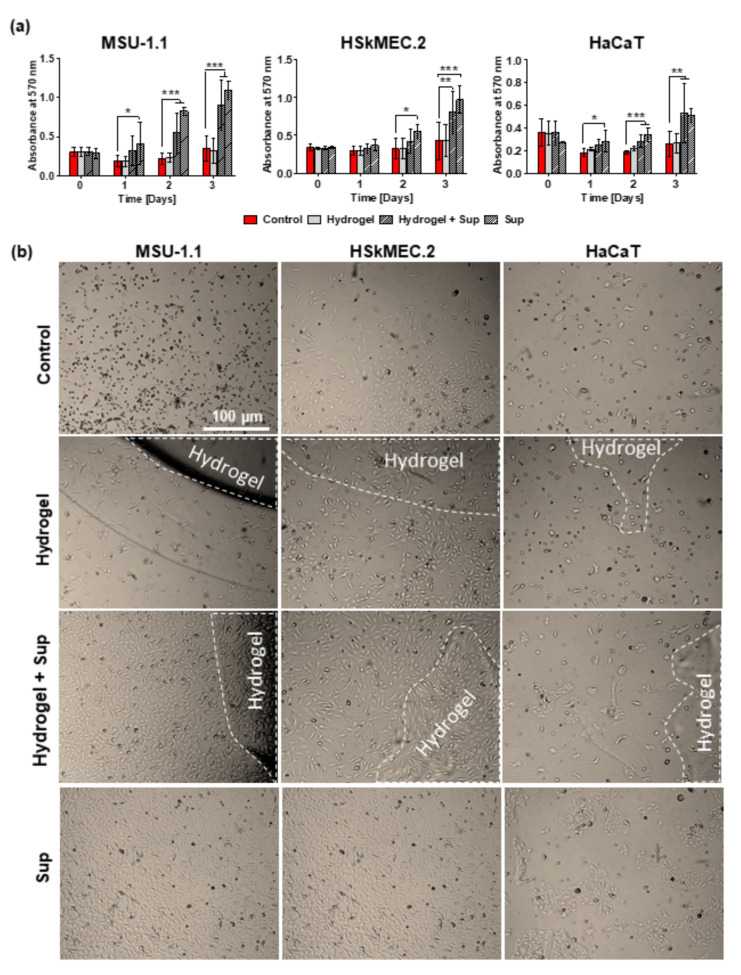
Proliferative activity of skin-derived cells under hydrogel-released HATMSC2 trophic factors. (**a**) Metabolic activity of MSU-1.1, HSkMEC.2 and HaCaT cells cultured in serum-free medium and 1% O_2_ was measured by MTT assay at day 0, 1, 2 and 3 following treatment with empty hydrogel, supernatant-loaded hydrogel and 22 µg HATMSC2 supernatant. Untreated cells were used as a control. Data represent mean ± SEM, *n* = 3, * *p* < 0.05, ** *p* < 0.01, *** *p* < 0.001. (**b**) Representative images of MSU-1.1, HSkMEC.2 and HaCaT alone, treated with empty hydrogel, supernatant-loaded hydrogel and 22 µg HATMSC supernatant protein following three-day culture in serum-free medium and 1% O_2_. Areas of the wells covered by the hydrogel are marked by a dashed line.

**Figure 5:**
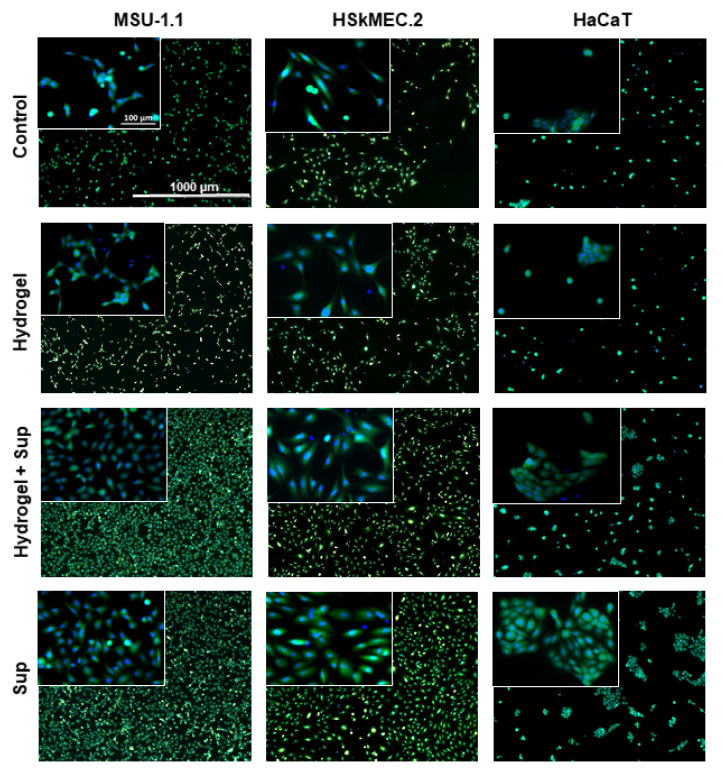
Proliferative activity of skin-derived cells under hydrogel-released HATMSC2 trophic factors measured by Live/Dead assay. Fluorescent images (calcein-green and DAPI-blue) of MSU-1.1 (LH panel), HSkMEC.2 (middle panel) and HaCaT (RH panel) following three-day culture in serum-free medium and 1% O_2_ (untreated controls) and cells treated with empty hydrogel, supernatant-loaded hydrogel and 22 µg HATMSC2 supernatant protein. Inserts on the left top corners are 10× magnifications of the original images.

To prove the biological activity of hydrogel loaded with HATMSC2 supernatant, the proliferation of fibroblasts (MSU-1.1), keratinocytes (HaCaT), and endothelial cells (HSkMEC.2) was evaluated during a 3-days culture in an in vitro model of wound. The results of MTT assay (Figure 4a) indicated an increase in all skin-derived cells metabolic activity in the presence of a previously optimized dose of 22 µg supernatant-present proteins alone or hydrogel loaded with an equal amount of supernatant proteins. However, the metabolic activity of cells treated with supernatant was usually higher than that of cells treated with supernatant-loaded hydrogels (though not significant). This was most probably because not all trophic factors were released from hydrogels which were visible in cell cultures until the last day of the experiment (Figure 4b), therefore the final concentration of biologically active factors in the cell culture medium was lower in these groups. In contrast to the untreated control groups, the supernatant delivered to the cell culture in the hydrogel carrier already showed significant biological activity on day 2 of treatment in the case of MSU-1.1 and HaCaT cells (*p* < 0.001). The highest metabolic activity of all cells treated with supernatant-loaded hydrogels was observed for the following three days, while no increase in cell proliferation was observed in untreated controls or unloaded hydrogel-treated cells. The increase in the metabolic activity was correlated with the number of live cells which was confirmed by bright-field microscopy observation (Figure 4b) and Calcein-AM/DAPI staining of the living cells after 3-day (Figure 5). The groups treated with supernatant alone showed a level of metabolic activity similar to that of groups treated with supernatant loaded hydrogels.

### 2.4. Release of HATMSC2 Supernatant-Present Proteins from Hydrogel

**Figure 6:**
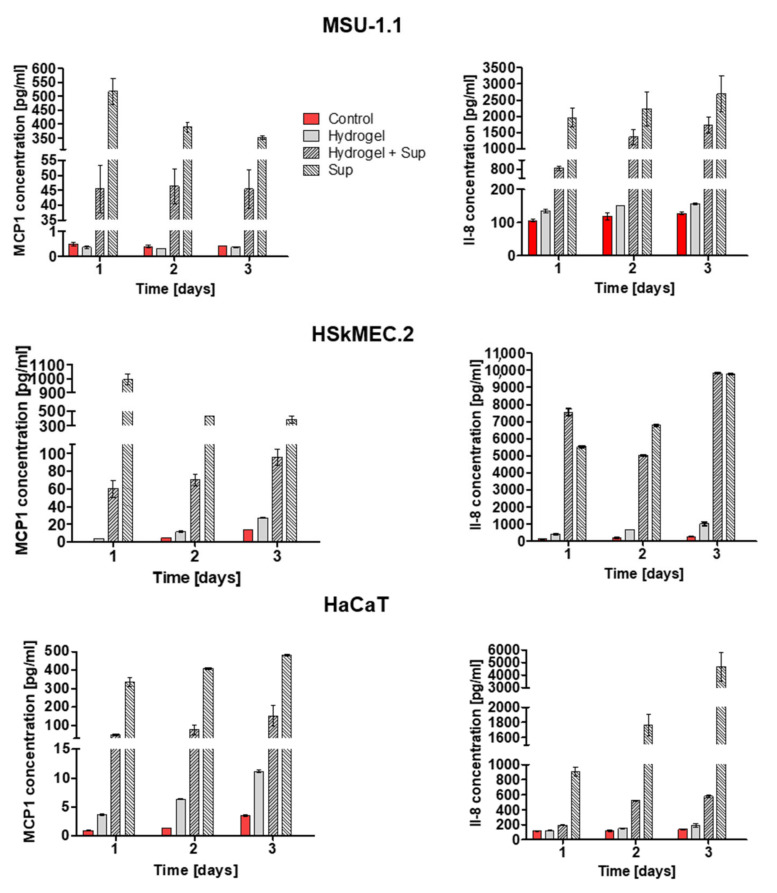
Release of HATMSC2 supernatant-present proteins MCP-1 and IL-8 measured by ELISA. The concentration of MCP-1 (LH panel) and IL-8 (RH panel) measured in culture medium collected from MSU-1.1, HSkMEC.2 and HaCaT cells alone, treated with empty hydrogel, supernatant-loaded hydrogel and 22 µg supernatant following 1, 2 and 3 days culture in serum-free medium and 1% O_2_. Data represent pulled values from three independent experiments with mean ± SD from two technical repeats.

Our previous work documented that the HATMSC2 supernatant contains a high level of interleukin-8 (IL-8) and macrophage chemoattractant protein-1 (MCP-1) [10]. To continue the assessment of bioactivity of the supernatants loaded in the hydrogel, the same trophic factors were selected to check the protein release from hydrogel following 1, 2 and 3 days (Figure 6). In all tested cell lines, the highest concentrations of both proteins were found in supernatant–treated samples and supernatant-loaded hydrogels samples; however, the protein concentration in the groups treated with the supernatant alone was higher than that in the groups treated with the hydrogel containing the supernatant. For example, for MSU-1.1 cells, the level of MCP-1 and IL-8 in supernatant-treated group were 517 pg/mL and 1936 pg/mL, while in cells treated with supernatant-loaded hydrogel, the level MCP-1 and IL-8 were 45 pg/mL and 135 pg/mL, respectively. It is important to highlight that the in vitro release of MCP-1 and IL-8 was measured in cell–containing systems (MSU-1.1, HSkMEC.2, and HaCaT), thus a part of the detected proteins came from the added/released supernatant and a part was produced by the cells. However, for this research, the most important observation is a substantial difference in the level of selected proteins between unloaded hydrogels and supernatant–loaded hydrogels groups. For example, for MSU-1.1 cells the levels of MCP-1 and IL-8 in hydrogel treated groups were 0.4 pg/mL and 135 pg/mL on day 1, and 0.4 pg/mL and 156.2 pg/mL on day 3, respectively. However, in MSU-1.1 cells treated with supernatant-loaded hydrogel the level of MCP-1 and IL-8 were much higher, 45 pg/mL and 810 pg/mL on day 1, respectively, and 45.5 pg/mL and 1723.7 pg/mL on day 3, respectively. A similar trend was observed in HaCaT and HSkMEC.2 cells where the levels of detected proteins were always higher in supernatant–loaded hydrogels groups than in unloaded hydrogel groups, and their concentrations rose on day 3. These results confirm that trophic factors are released from the hydrogel enriched with HATMSC2-derived supernatant as early as day one and their levels were maintained until the third day.

### 2.5. Pro-Angiogenic Activity of Hydrogel-Released HATMSC2-Originated Trophic Factors

**Figure 7:**
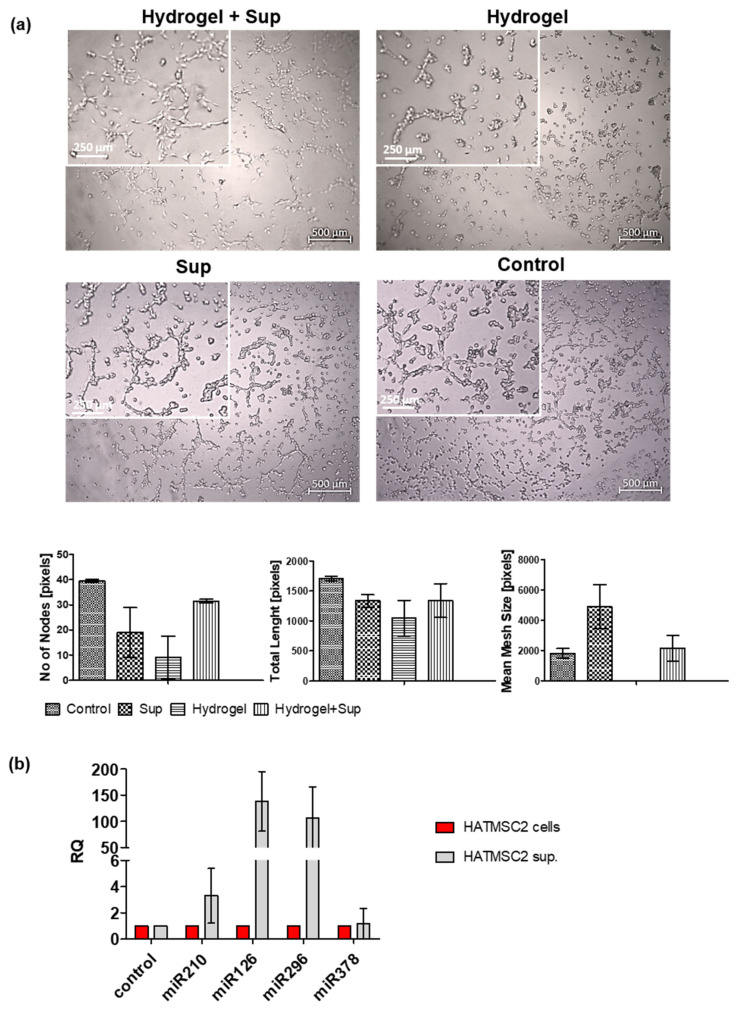
Pro-angiogenic activity of hydrogel-released HATMSC2 supernatant. (**a**) Representative images of in vitro angiogenesis of skin endothelial cells (HSkMEC.2) following 22h culture in the presence of supernatant-loaded hydrogel or supernatant treatment alone. The supernatant alone group (Sup) and Matrigel control (Control) are our positive controls. Graphs present quantification of angiogenesis by the number of nodes, total length of the tubes and mesh size. Inserts on the left top corners are 2.5 x magnifications of the original images. Data represent as mean ± SD, *n* = 2; (**b**) Relative expression of proangiogenic miRNAs, miR-210, miR-126, miR-296, and miR-378 in HATMSC2 supernatant. The expression of miRs was determined in a real-time RT-PCR experiment and measured relative to the control (HATMSC2 cells). Data were presented as mean ± SD values, *n* = 2.

The biological activity of hydrogel loaded with HATMSC2 supernatant was also investigated using the in vitro tube formation assay. Human skin endothelial cells (HSkMEC.2) were seeded into a 96-well plate, coated with growth factor-reduced Matrigel, in the presence of supernatant-loaded hydrogel or unloaded hydrogel (Figure 7a top panel). As a control, HSkMEC.2 cells were seeded on Matrigel without hydrogel in the presence or absence of HATMSC2 supernatant (Figure 7a bottom panel). Tube formation in the unloaded hydrogel was less effective than in supernatant treated or untreated controls. However, supplementation of hydrogel with supernatant improved angiogenic properties of HSkMEC.2 as evidenced by the formation of loops by these cells. This result suggests that trophic and pro-angiogenic factors were released from the hydrogel loaded with HATMSC2 supernatant and that its pro-angiogenic properties were preserved. The pro-angiogenic properties of HATMSC2 supernatant were also confirmed by the expression of pro-angiogenic miRNAs (Figure 7b). The expression of selected proangiogenic regulatory molecules such as miR210, miR126, miR296 and miR378 was analyzed in both HATMSC2 cell line and HATMSC2 supernatant. It was found that the relative expression of miR210, miR126 and miR296 was higher in HATMSC2 supernatant than in the HATMSC2 cells. The highest relative expression was observed for miR126, RQ = 130 (Figure 7b).

### 2.6. Antimicrobial Activity of HATMSC Supernatant

**Figure 8:**
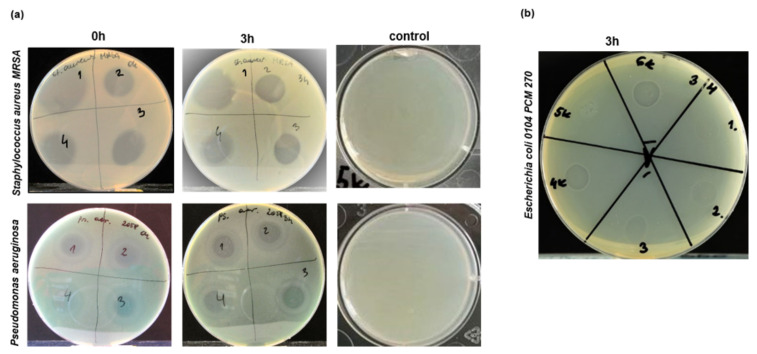
Antimicrobial activity of HATMSC supernatants. (**a**) Growth inhibition of *S. aureus* (top panel) and *P. aeruginosa* (bottom panel) following treatment with supernatants collected from human adipose tissue MSC cell lines: 1—HATMSC1, 2—HATMSC2, 3—HATMSC2D10 and HATMSC2F10. Supernatant samples were applied immediately following bacteria application on plates (0h) or three hours later (3 h). As a control, the DMEM medium without antibiotic used for supernatant production was applied; (**b**) Growth inhibition of *E. coli* following treatment of hydrogel loaded with HATMSC2 supernatant: 1—1:1 (*v*/*v*), 2—1:2 (*v*/*v*), 3—1:3 (*v*/*v*), and controls 4—supernatant only, 5—hydrogel only, 6—DMEM with supernatant 1:3 (*v*/*v*).

*Staphylococcus aureus* and *Pseudomonas aeruginosa* are the most common and difficult to clear out pathogens found in the wounds [15,16], therefore antimicrobial therapy would improve the treatment efficiency of chronic wound. To investigate whether antibacterial effects can be obtained by HATMSCs supernatants, a pilot experiment was carried out where supernatants collected from HATMSC2, and previously generated HATMSC1, HATMSC2D10, and HATMSC2F10 cell lines [10] were compared for their antibacterial activity. Results of the antimicrobial activity experiment of all studied supernatants are presented in Table 1, and representative images presenting growth inhibition of Gram-positive bacteria *Staphylococcus aureus* MRSA and Gram-negative bacteria *Pseudomonas aeruginosa* are shown in Figure 8a. Complete inhibition of growth (+++) was observed in the case of bacterial strains belonging to Gram-positive bacteria such as *Staphylococcus aureus* (including methicillin-resistant *Staphylococcal aureus* (MRSA) PCM 3144, PCM 519 and Covan 2101) and *Staphylococcus epidermidis* PCM 2651, and for Gram-negative bacteria such as *Escherichia* *coli* (PCM 270, PCM 2144). Application of the supernatant samples immediately after loading the bacteria (0h) and 3 h later (3h) had the same result. Weaker inhibitory effect on the growth of bacteria (++) was observed in the case of *Pseudomonas aeruginosa* PCM 2270 while the least sensitive to supernatants treatment was *Pseudomonas aeruginosa* PCM 2058 where weak bacterial growth inhibition (+) was observed. In contrast, proper growth of all tested bacterial strains was observed following treatment with DMEM medium without antibiotics used as a control. To prove that antimicrobial activity of the HATMSC2 supernatant is preserved following incorporation into hydrogel, mixtures of hydrogel with the supernatant at different *v*/*v* ratios (1:1, 1:2 and 1:3) were tested on *Escherichia* *coli* (PCM 270) (Figure 8b). A weak inhibition of bacteria growth was observed for 1:2 ratio, whereas moderate bacterial growth inhibition was seen in the case of 1:3 *v*/*v* hydrogel to supernatant ratio was applied.

## 3. Discussion

The research related to the use of MSCs in the treatment of difficult-to-heal wounds has evolved over the years, starting with autologous cell transplants [17], transplantation of cells embedded in hydrogels [18,19], through the delivery of a MSC secretome as a whole [3] or isolated exosomes [20,21]. The latest trends in research on cell-free therapy focus on the use of MSC-produced bioactive factors in combination with hydrogel carriers. This dual-approach that combines the advantages of cell therapy and hydrogel dressing but abolishes the limitation related to MSC transplantation such as procedure severity, limited cell survival, donors’ dependent alterations in cell phenotype, differentiation and secretion potential, seems to be a very promising solution. Therefore, in this research, we went a step further and propose an innovative wound dressing consisting of active factors produced by recently established by our research group human adipose tissue derived MSC cell lines [10] embedded in the collagen hydrogels.

Collagen-based wound dressings have been widely used over many years because they enhance and influence wound healing [22,23]. Collagen polymers promote tissue granulation and angiogenesis while inhibiting bacterial growth and prolonged inflammatory response [24]. Collagen hydrogels are also frequently used as platforms for drug delivery due to their low immunogenicity, biocompatibility, and similarity to the natural extracellular matrix (ECM) [25]. In this study for hydrogel preparation, we used collagen type 1, which is the most common form of collagen found in connective tissues including skin, and cross-linked it using 10K 4-arm Succinimidyl Glutarate PEG (4ARM-SG-10K). The cross-linker used did not exhibit any toxicity to fibroblasts, endothelial cells or to keratinocytes when compared to standard 2D tissue culture controls. Consistent with our study, collagen cross-linking with 4-arm PEG succinimidyl glutarate did not affect dermal fibroblast viability [26,27]. Our results suggested that the composed hydrogel is biocompatible with living cells and suitable for further in vivo examination. Having in mind a potential use of the hydrogel as a wound dressing, which would ideally be changed once or twice weekly, we have looked at the degradation profile for up to 6 days. We observed that hydrogels reduced their mass to 40 and 30 percent of the initial mass on day 3 and day 6, respectively. Total in vitro hydrolytic degradation for hydrogels composed of collagen and 4ARM-SG is observed within 5–6 weeks [28]. In contrast, upon addition of collagenase, the hydrogels readily degraded within 1 day and this was consistent with other studies where enzymatic degradation was observed within hours [29]. However, increasing hydroxyproline concentration was evident in the remaining degradation buffer for the next six days suggesting that although hydrogels are not visible by to the eye, its degradation continues nevertheless. All of this suggests that the hydrogel used in this study is appropriate as a carrier for HATMSC2-originated trophic factors because due to its degradation, which will be even increased in vivo by a number of factors such as presence of metalloproteinases and inflammatory cells, will result in the release of active factors to the target tissue.

The main goal of this research was to confirm that HATMSC2s-produced factors maintained their biological activity following incorporation into the collagen hydrogel and subsequent release. To investigate this, we compared the effects of HATMSCs supernatant treatment to those of supernatant-loaded hydrogel and unloaded hydrogel on cell metabolic activity, proliferation and angiogenic properties. Hydrogel may act as a reservoir of therapeutic factors which are being released in a sustained manner. It has been reported that controlled release of several factors such as epidermal growth factor [30,31,32] stromal cell-derived factor-1 [33] or fibroblast growth factor-2 [34] has been achieved from hydrogel systems and, to some extent, promoted the wound-healing process in animal models of the disease. Gel eluting recombinant human platelet-derived growth factor has been tested in clinical studies [35]. However, application of the singular recombinant factor is less effective than the synergistic activity of natural growth factors and a cytokines cocktail produced by atMSCs. To date, only a few studies have investigated the therapeutic effect of the hydrogels containing MSC-conditioned medium on wound healing. It has been demonstrated that human umbilical cord MSC-conditioned medium in collagen-based hydrogels promoted burn injury healing in mice [36,37]. Our previous studies indicated that the supernatant harvested from established HATMSC2 cell line in in vitro settings acts better than MSCs alone or supernatant harvested from primary cells, and thus has a very high clinical implementation potential [10]. In this study, we confirmed the hypothesis that the therapeutic effect of HATMSC2-produced bioactive factors may be maintained following incorporation into biomaterial hydrogel. This was verified by the release of IL-8 and MCP-1 from the hydrogel loaded with supernatant and improved metabolic activity, as well as proliferation of fibroblasts, endothelial cells and keratinocytes in an in vitro model of chronic wounds. We conducted the experiment for three days, assuming that the potential wound dressing would be changed at that time. Our previous results indicated that the supernatants collected from established HATMSCs lines contain a variety of proteins related to the regulation of angiogenesis [10]. Recently, we have also documented that microvesicles from HATMSC1 cell line of adipose tissue origin carry proteins and miRNAs that support and facilitate angiogenic processes [38]. In this study, we have extended the research on the pro-angiogenic properties of the HATMSC2 supernatant, including analysis of the presence of proangiogenic regulators miRNAs. The reduced angiogenic ability contributes to impaired wound healing and therefore pro-angiogenic therapy is highly desirable. It was shown that topical application of conditioned medium from induced pluripotent stem cells [39] or hydrogel-mediated delivery of umbilical cord blood-derived MSC [36] accelerates wound healing through enhanced angiogenesis. Our results clearly show that the supplementation of hydrogel with HATMSCs supernatant improves the tube formation process in angiogenic test in vitro. Moreover, we have confirmed the expression of four pro-angiogenic miRNA in the HATMSC2 supernatant. The expression of miR210, miR126 and miR296 was higher in HATMSCs supernatant than in intact cells indicating that HATMSCs supernatant can support proangiogenic processes in tissue regeneration. These results suggest the possible beneficial effect of HATMSCs supernatant-loaded hydrogel dressing on the wound healing process in the context of restoration of proper angiogenesis.

An ideal wound dressing should also counteract skin infections, which often accompanies chronic wounds. Therefore, we have finally investigated whether supernatants harvested from the established HATMSCs cell lines have antimicrobial properties. A pilot screening against the most common pathogens found in the wounds, such as *Staphylococcus aureus, Staphylococcus epidermidis*, or *Pseudomonas aeruginosa* demonstrated the antimicrobial potential of all HATMSCs supernatants and no differences were observed in antibacterial activity between all four tested supernatants. This was consistent with other studies where MSC-conditioned medium revealed antibacterial activity when tested in vitro [12,40,41] or in an in vivo model of chronic wounds [42]. We have also shown that antibacterial activity of the HATMSC2 supernatant was preserved following incorporation into hydrogel what was proved by *E. coli* growth inhibition. Interestingly, during our search of the literature, no information was found on established MSC cell lines with such antibacterial properties. Although not investigated in this study, it could be assumed that antimicrobial activity of the HATMSCs supernatants is mediated through antimicrobial peptides and proteins (AMPs) which can inhibit the growth of bacteria. AMPs or host-defense peptides and proteins are an abundant and diverse group of endogenous molecules that are produced as a first line of defense by all multicellular organisms which have a broad spectrum of antimicrobial and immunomodulatory activities [43]. It was reported that MSCs can inhibit bacterial growth by secretion of AMPs [11,12], therefore a more comprehensive analysis of antibacterial properties is needed. Nevertheless, at this stage, it can already be concluded that the HATMSC2 supernatant also has the potential for antibacterial activity.

## 4. Materials and Methods

All reagents used in this study were purchased from Merck, Poznan, Poland Ltd. unless otherwise stated. All tissue culture materials were purchased from BD Biosciences, Warsaw, Poland unless otherwise stated.

### 4.1. HATMSC Supernatant Preparation

HATMSC supernatant was prepared as described previously [10] with minor changes. Briefly, HATMSCs were seeded in a T500 flask (Thermo Scientific, Roskilde, Denmark) at the density of 1.9 × 10^4^ cells per cm^2^ in DMEM, 10% Human Serum (HS). Following 24 h incubation at 37 °C, 5% CO_2_, the culture medium was removed from the flask, and the culture dish was washed and replaced with DMEM without serum. Following 24 h culture under hypoxic conditions (1% O_2_, 5% CO_2_), conditioned medium was collected and centrifuged for 10 min, 300 g to remove cellular debris. Collected native HATMSCs supernatants were concentrated first 5-fold (*v*/*v*) with 3 kDa Filters Amicon^®^ Stirred Cells 400 mL (EMD Millipore Corporation, Massachusetts, MA, USA) followed by further concentration using Amicon^®^ Ultra 15 mL Centrifugal 3 kDa Filters (Merck Millipore, Carrigtwohill, Ireland) which resulted in an approximately 10-fold (*v*/*v*) enrichment. Total protein concentration in the native and concentrated supernatants was measured based on the method of Bradford using a Bio-Rad Protein Assay (Bio-Rad, Munich, Germany). Supernatants were stored at 4 °C before use.

### 4.2. Hydrogel Formation

To obtain 500 μL of collagen hydrogel, 50 μL of five times concentrated PBS was added to 166.6 μL of 6 mg/mL type I collagen solution (Collagen Solutions, Glasgow, UK) and mixed by gentle pipetting. Next, to neutralize the acidic collagen solution, 2 μL of 1M NaOH was added and mixed thoroughly by pipetting. Collagen solutions were then mixed by gentle pipetting with 225 µL HATMSC supernatant (1 mg/mL) or an equal volume of PBS buffer (empty hydrogel). Prior to use, the cross-linker 10K 4-arm Succinimidyl Glutarate PEG (4ARM-SG-10K) (JenKem Technology, Beijing, China) was dissolved in a miliQ water to its final concentration of 19.6 mg/mL. In the last step, 56.4 µL of the cross-linker solution was added to allow the active ester groups to react with the free amino groups of collagen. Following gentle mixing, the hydrogel was immediately pipetted onto a sterile parafilm in a petri dish into 50 μL aliquots. Closed dishes containing hydrogels were incubated for one hour at 37 °C to allow complete cross-linking.

### 4.3. Cytotoxicity of Hydrogel

The potential cytotoxicity of the hydrogel was evaluated on human fibroblast cell line MSU-1.1 [44], human keratinocytes cell line (HaCaT) purchased from the DKFZ collection, and normal human skin microvascular endothelial cells (HSkMEC.2) obtained and patented by our research group in cooperation with the Centre National de la Recherche Scientifique, France, (patent 99–16169), as previously described [45]. MSU-1.1 and HaCaT cells were cultured in DMEM, 10% FBS, 1% pen/strep while HSkMEC.2 were cultured in Opti-MEM with GlutaMAX supplemented with 2% Fetal Bovine Serum (FBS, HyClone, UK) and 1% pen/strep. Cells were seeded in a 24-well plate at the density 1.25 × 10^4^ (MSU-1.1 and HSkMEC.2) or 2.5 × 10^4^ (HaCaT) cells per well in 400 μL of culture medium. Following 2 h at 37 °C, 5% CO_2_, 50 μL hydrogel spheres were added to the wells using a sterile spatula. Cells cultured without hydrogels were used as controls. Potential cytotoxicity of hydrogels was evaluated by metabolic activity (MTT assay) at time 0 and following 1, 2 and 3 days. Briefly, at the desired time point culture, medium and hydrogels were removed from wells and replaced with 400 μL of fresh medium supplemented with 10% MTT (Sigma-Aldrich, cat# M5655, St. Louis, MO, USA). Following 4 h incubation at 37 °C, 5% CO_2_ medium was removed, and cells were solubilized with 300 µL of DMSO per well and absorbance was measured at 570 nm.

### 4.4. Hydrogel Degradation

In vitro degradation of 50 μL hydrogel spheres was monitored in the presence or absence of collagenase enzyme. The prepared collagen hydrogels were exposed to the solution of collagenase at 37 °C for 6 days. Enzyme solution was made by dissolving collagenase from Clostridium histolyticum (Type I-A) in DMEM medium and frozen at −20 °C. Finally, the collagenase solution was diluted in PBS to a concentration of 25 CDU (collagen digestion unit)/mL. Next, 400 μL of this enzyme solution was added to 50 μL of collagen hydrogel in a well of 24-well plate at 37 °C. As a control, the same volume of PBS buffer was added to 50 μL of collagen hydrogel. At the given time point (1–6 days), supernatant was transferred to an Eppendorf tube and stored at −20 °C for further analysis. In control samples without collagenase, the hydrogels removed from the solution were weighed.

### 4.5. Hydroxyproline Assay

The hydroxyproline concentration in the collected supernatants was determined using a Hydroxyproline Assay Kit according to the manufacturer’s protocol. Briefly, supernatant samples were hydrolysed at a final concentration of 6 M hydrochloric acid at 120 °C for 3 h. Then, 4 mg of activated charcoal was added to each sample and samples were centrifuged at 10,000× *g* for 3 min. Following this, 30 μL of samples and 0–10 μL of hydroxyproline standard solution were transferred to a 96 well-plate which was placed into a 60 °C oven to dry samples. Then 100 μL of freshly prepared Chloramine T/Oxidation Buffer was added to each well and plate was incubated for 5 min at RT. Following the addition of 100 μL of DMAB Reagent to each sample and standard well, the plate was incubated for 90 min at 60 °C, and absorbance was measured at 560 nm. The percentage of hydroxyproline release was calculated, where hydrogel from day 0 was used as 100%.

### 4.6. Bioactivity of the Released Trophic Factors from Hydrogel Loaded with Supernatant

The biological activity of hydrogel-released HATMSC2 supernatant was evaluated using an in vitro model of chronic wound as described previously [10] with minor changes. Briefly, cells were seeded in a 24-well plate at the density 1.25 × 10^4^ (MSU-1.1 and HSkMEC.2) or 2.5 × 10^4^ (HaCaT) cells per well in 400 μL DMEM—serum-free medium. Following 2 h at 37 °C, 5% CO_2_, 50 μL supernatant-loaded or unloaded hydrogel spheres were added to the wells. Cells cultured without hydrogels and cells treated with 22.5 µg of HATMSC2 supernatant proteins were used as controls. Cells were cultured under hypoxic conditions (1% O_2_) and following 0, 1, 2 and 3 days gels were removed from the wells and metabolic activity was measured by the MTT assay. In parallel, cells were seeded in 24-well plates and treated in the same manner for LIVE/DEAD™ Cell Imaging Kit (Thermo Fisher, Massachusetts, MA, USA). On the third day of culture under hypoxic conditions, the working solution of calcein (live) and ethidium homodimer (dead) was prepared according to the manufacturer’s protocol and 200 μL of this mixture was added into each well and incubated for 15 min at 25 °C. Before cell imaging acquisition, DAPI was added into each well. Images were obtained using an Axio Observer inverted microscope equipped with a dry 5x lens (Zeiss, Gottingen, Germany). The images were processed with the Zen Blue software (Zeiss, Gottingen, Germany).

### 4.7. Supernatant Release Study

Supernatant in vitro release profile was characterized by IL-8 and MCP-1 ELISAs. MSU-1.1, HSkMEC.2 and HaCaT cells were seeded in a 24-well plate in 400 μL DMEM—serum-free medium and treated with empty or supernatant-loaded hydrogel spheres while untreated cells and cells treated with a previously optimized dose of 22.5 µg of HATMSC2 supernatant were used as controls. Cells were cultured under hypoxic conditions (1% O_2_) and, following 24 h, cell culture medium was collected and frozen at −20 °C until ELISA was conducted. The level of supernatant components released from hydrogels was measured using Human IL-8/CXCL8 ELISA Kit and Human MCP-1/CCL2 ELISA Kit (Sigma-Aldrich, Missouri, MO, USA) according to the manufacturer’s protocol. ELISAs were performed using pulled cell culture medium samples from three independent experiments, with two technical repeats.

### 4.8. In Vitro Angiogenesis in Matrigel Matrix

The pro-angiogenic potential of HATMSC2 supernatant-loaded hydrogel was tested by the tube formation assay using HSkMEC.2 endothelial cells. Fifty μL of collagen hydrogels with 22.5 µL HATMSC2 supernatant or PBS were pipetted into a 96-well plate and incubated for 1h at 37 °C to allow gel polymerization. Next, hydrogels were covered with 50 µL of growth factor-reduced Matrigel (BD Bioscience, Massachusetts, MA, USA) while control wells were coated only with Matrigel. Cells were seeded at the density of 1.5 × 10^4^ cells per well in serum-free DMEM with or without supernatant. Cell culture was monitored under an inverted light microscope every 2 h, until pseudovessels were completely formed. Angiogenesis was calculated using the ImageJ Angiogenesis Analyzer software by measurement of mean mesh size, the number of nodes, and total length of the tubes.

### 4.9. Examination of MicroRNAs Present in HATMSC Supernatant

The presence of four angiomiRs (miR-126, miR-296, miR-378, and miR-210) in HATMSCs supernatants was investigated using real-time RT-PCR with the TaqMan technique. Total cellular RNA was isolated from HATMSC2 cells using the NucleoSpin RNA kit (MACHEREY-NAGEL, Düren, Germany). The isolation of total RNA from HATMSC2 supernatants was performed according to Glynn et al. [46]. Briefly, 1 mL of each supernatant was treated with Trizol reagent and chloroform, centrifuged at 15–300× *g* (15 min., 4 °C) and the aqueous phase was then subjected to isolation using the NucleoSpin RNA kit. First-strand cDNA was synthesized through a reverse transcription of 7.5 ng of total RNA using the TaqMan MicroRNA Reverse Transcription Kit (Thermo Fisher Scientific Inc., Waltham, MA, USA). The reverse transcription was carried out with microRNA primers for angiomiRs: miR-210 (assay ID 000512), miR-126 (assay ID 002228), miR-296 (assay ID 002101), miR-378 (assay ID 001314), and RNU48 as an internal control (assay ID 001006). Real-time PCR was then performed with the abovementioned microRNA primers in the ViiA7 Real-Time PCR System according to the TaqMan Small RNA Assays Protocol. The expression of the analyzed miRs in supernatants was calculated relative to the controls (HATMSC2 cells).

### 4.10. Bacterial Growth and Antimicrobial Activity of HATMSC Supernatants

Eight bacterial strains from the Polish Collection of Microorganisms (PCM) at the Institute of Immunology and Experimental Therapy, PAS, were used for this research including Gram-positive bacteria such as *Staphylococcus aureus* MRSA PCM 3144; *Staphylococcus aureus* PCM 519; *Staphylococcus aureus* Covan PCM 2101; *Staphylococcus epidermidis* PCM 2651 and Gram-negative bacteria such as *Escherichia coli* O104 PCM 270; *Escherichia coli* PCM 1144; *Pseudomonas aeruginosa* PCM 2270*; Pseudomonas aeruginosa* PCM 2058. The antimicrobial activity of HATMSC supernatants was tested by the standard procedure used in the determination of bacterial susceptibility to bacteriophage action [47,48]. Briefly, 24 h-culture of each bacterial strain in the liquid medium enriched with glucose with optical density (OD) of 1.0 was applied onto a dried agar plate. An excess of bacteria culture was removed in a sterile manner from the plate and the plate was incubated for approximately 20–30 min at 37 °C to dry the excess liquid. Then 40 µL of HATMSCs supernatant samples (1mg/mL) or 40 µL DMEM were applied to each agar plate with a single bacterial strain immediately after drying the plate (0 h) or following 3 h incubation of bacteria on the plate at 37 °C (3 h). After 24 h, photos of plates were taken and antimicrobial activity of HATMSC supernatants was visually evaluated based on an estimation of bacteria growth inhibition. In addition, activity of the released antibacterial factors from hydrogel loaded with HATMSC2 supernatant was tested on Escherichia coli O104 PCM 270 following 3 h incubation of bacteria on the plate at 37 °C. Hydrogel was mixed with the HATMSC2 supernatant (1 mg/mL) in the following ratios (*v*/*v*): 1:1, 1:2, 1:3 and 40 µL of this mixture was applied to the bacteria-seeded agar plate. As controls, 40 µL of hydrogel only, supernatant (1 mg/mL), and DMEM with supernatant 1:3 (*v*/*v*) was applied. Quantification of the obtained results were as follows: (+++)—positive results—complete bacterial growth inhibition; (++)—positive results—moderate bacterial growth inhibition; (+)—a weak bacterial growth inhibition; (-)—negative result—absence of growth inhibition.

### 4.11. Statistical Analysis

Statistical analyses were performed using GraphPad Prism version 7 (GraphPad Software, California, CA, USA.). Data were compared using one-way analysis of variance (ANOVA) followed by multiple comparison procedures (Dunnet’s test). Values were considered to be significantly different with a *p* < 0.05.

## 5. Conclusions

The results of this study indicate that the developed collagen hydrogel loaded with HATMSC2 supernatant has pro-proliferative and pro-angiogenic activity while tested in an in vitro wound model. In addition, pilot data indicated that HATMSC2 supernatant also has antimicrobial properties. Taken together, this suggests that the developed hydrogel loaded with HATMSC2 bioactive factors has the potential to be used as an advanced wound dressing with trophic, immunomodulatory, antimicrobial and pro-regenerative properties. The results proved that hydrogel-loaded supernatant exert similar biological effect as supernatant alone and this suggests that the proposed hydrogel is appropriate as a carrier for the supernatant-present growth factors. Thus, hydrogel-loaded HATMSC2 supernatant can be a better solution as a drug formula for potential clinical use than supernatant alone. However, before this appealing idea can be introduced into the clinic, advanced research, including in vivo testing and thorough antimicrobial analysis needs to be done.

## Figures and Tables

**Table 1:** Characterization of antimicrobial activity of the HATMSC supernatants.

Bacterial Strains		HATMSC1		HATMSC2		HATMSC2D10		HATMSC2F10		Control	
		0 h *	3 h *	0 h	3 h	0 h	3 h	0 h	3 h	0 h	3 h
Gram-positive bacteria	*Staphylococcus aureus* MRSA PCM 3144	+++	+++	+++	+++	+++	+++	+++	+++	-	-
	*Staphylococcus aureus* PCM 519	++	+++	+++	+++	+++	+++	+++	+++	-	-
	*Staphylococcus aureus* Covan PCM 2101	+++	+++	++	+++	++	+++	++	+++	-	-
	*Staphylococcus epidermidis* PCM 2651	+++	+++	+++	+++	+++	+++	+++	+++	-	-
Gram-negative bacteria	*Escherichia coli* O104 PCM 270	+++	+++	+++	+++	+++	+++	+++	+++	-	-
	*Escherichia coli* PCM 1144	+++	+++	+++	+++	+++	+++	+++	+++	-	-
	*Pseudomonas aeruginosa* PCM 2270	++	++	++	++	++	++	++	++	-	-
	*Pseudomonas aeruginosa* PCM 2058	+	+	+	+	+	+	+	+	-	-

(+++)—completely bacterial growth inhibition; (++)—moderate bacterial growth inhibition; (+)—weak bacterial growth inhibition; (-)—no bacterial growth inhibition; (*)—time of application of HATMSCs supernatant following application of bacteria on agar plates.

## Data Availability

All data are included in this manuscript.
